# Supplementary figures and images for: Nuclear PYHIN proteins target the host transcription factor Sp1 thereby restricting HIV-1 in human macrophages and CD4+ T cells
Source: PLoS Pathog. 2020 Aug 6;16(8):e1008752. doi: 10.1371/journal.ppat.1008752 (PMC7433898; doi:10.1371/journal.ppat.1008752)

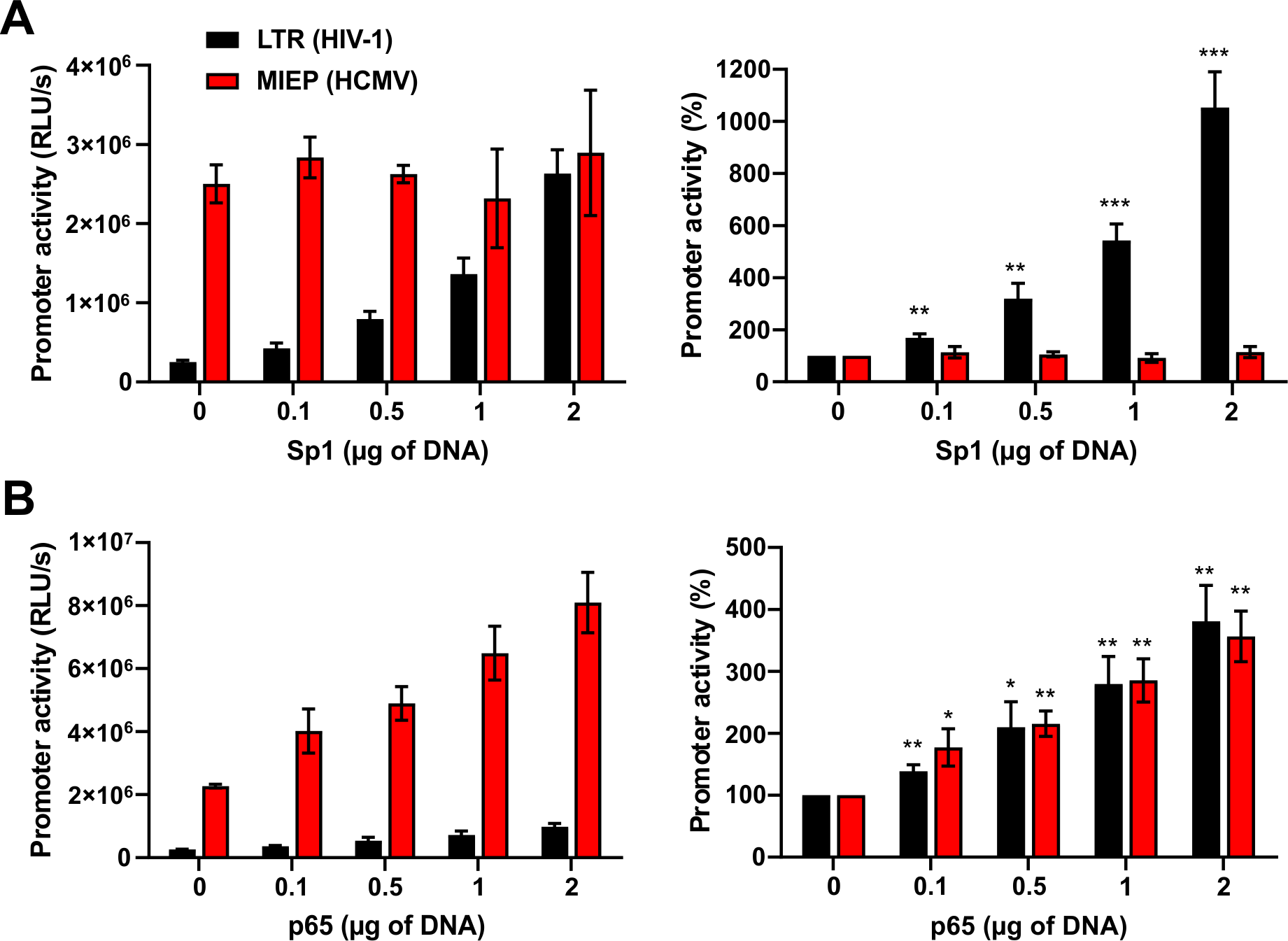

Supplement: S1 Fig — (A, B) HEK293T cells were cotransfected with luciferase reporter constructs under the control of the HIV-1 LTR (0.3 μg) or the CMV IE promoter (2 μg) and either an expression vector for (A) Sp1 or (B) p65, or a vector control. 48 hours post-transfection, luciferase activities were determined (n = 4 ±SD). * p < 0.05, ** p < 0.01, *** p < 0.001. (TIF) [file ppat.1008752.s001.tif]

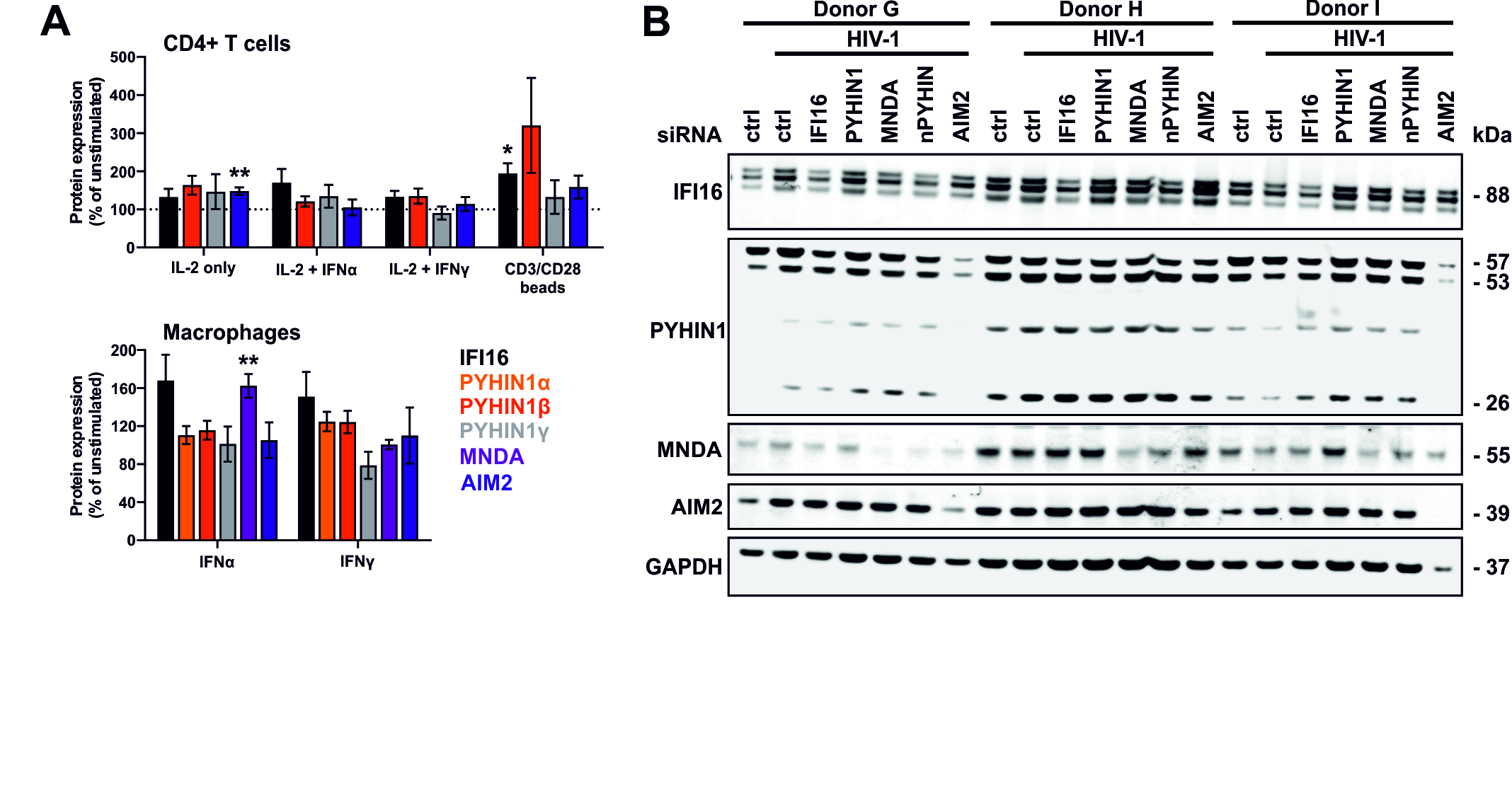

Supplement: S2 Fig — (A) CD4+ T lymphocytes or macrophages treated and analyzed by Western blot as described in the legend to Fig 3A. The bars show mean IFI16, PYHIN1, MNDA and AIM2 levels of three to five donors ±SEM. Calculations of the levels of PYHIN protein expression were always normalized to the GAPDH control. * p<0.05, ** p<0.01. (B) Western blot analysis of human MDM treated with PYHIN-specific or control siRNA. Shown is a representative example of three donors. Quantitative analyses are provided in Fig 3B. (TIF) [file ppat.1008752.s002.tif]

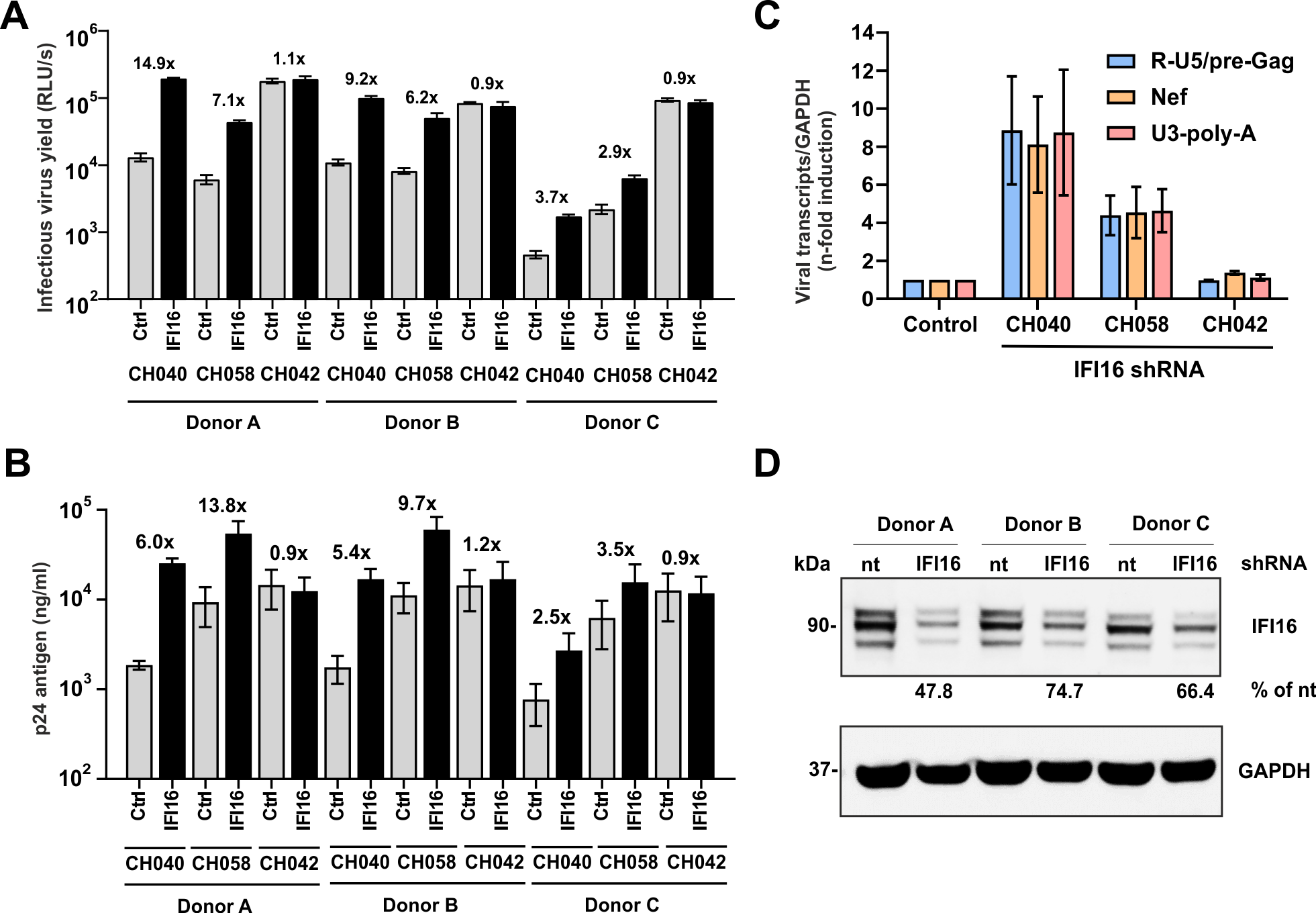

Supplement: S3 Fig — (A-D) CD4+ T cells were isolated, activated with IL-2 and anti-CD3/CD28 beads, treated with a mix of a control or an IFI16-targeting shRNA and transduced with the VSV-G pseudo-typed HIV-1 strains and infectious virus yield was assessed 72 hours later. Infectious virus yields (A), p24 antigen production (B), the levels of viral RNA transcripts (C) and IFI16 expression levels (D) were determined three days post-transduction. Numbers above bars indicate n-fold change between cells treated with control or IFI16 specific gRNA. (TIF) [file ppat.1008752.s003.tif]

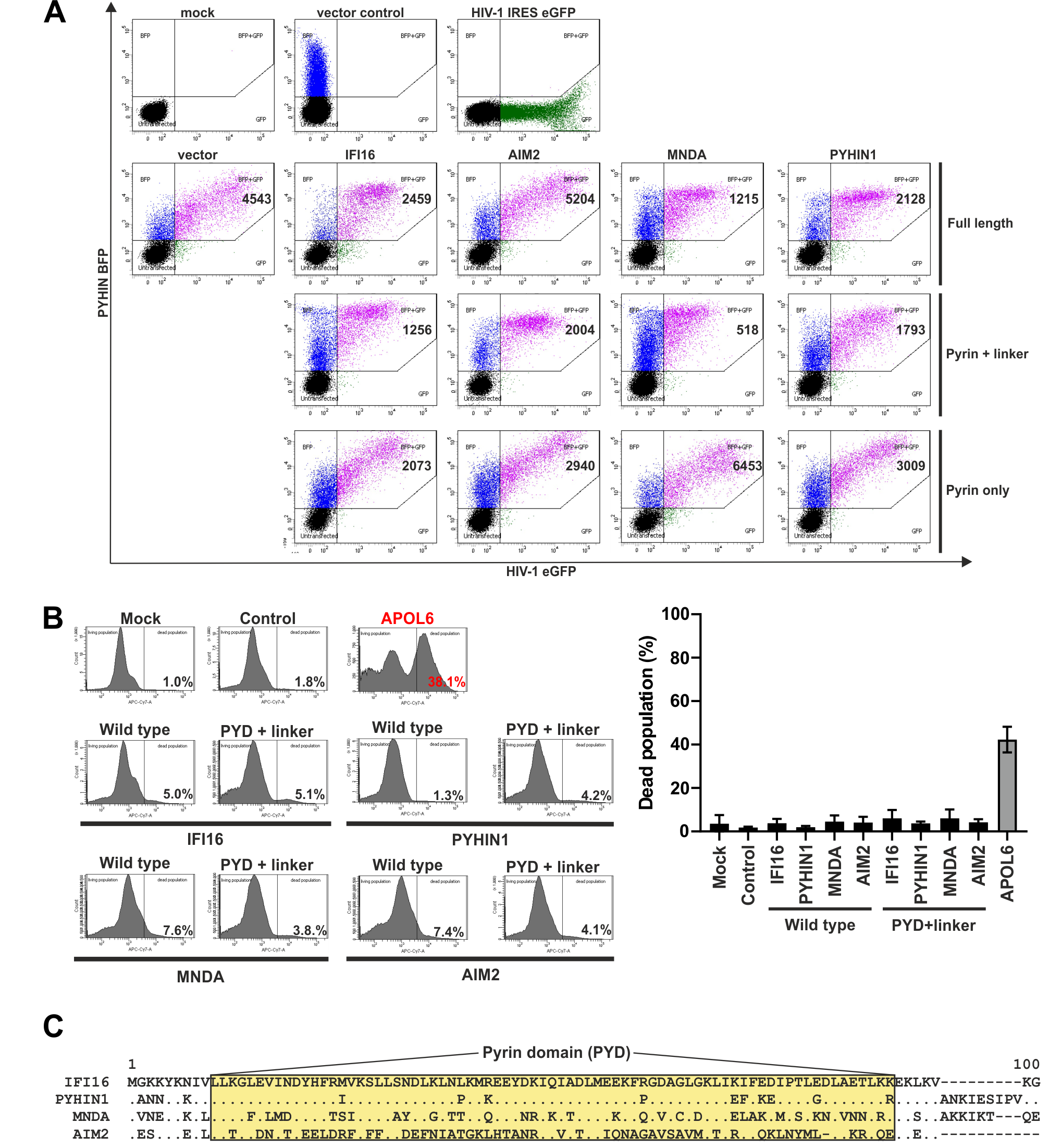

Supplement: S4 Fig — (A) HEK293T cells were cotransfected with HIV-1 NL4-3-IRES-eGFP and expression constructs for full length or mutants forms of PYHIN proteins. At 48 hours post transfection, cells were processed for FACS analysis and analyzed for eGFP and BFP expression. Numbers indicate eGFP MFI in the BFP+eGFP+ population. (B) Expression of PYHIN proteins does not cause cytotoxic effects. HEK293T cells were transfected with an empty vector or expression constructs for the indicated factors, harvested 48 hours later and stained with the Fixable Viability Dye eFluor 450 for flow cytometry. The living/dead population was assessed via FACS (n = 2–3 ± SD). A construct expressing APOL6 was used as a positive control. (C) Amino acid alignment of the N-terminal region of IFI16, PYHIN1, MNDA and AIM2. The shaded area highlights the PYDs, dots indicate amino acid identity and dashes gaps. (TIF) [file ppat.1008752.s004.tif]
