# Supplementary material for: Nuclear PYHIN proteins target the host transcription factor Sp1 thereby restricting HIV-1 in human macrophages and CD4+ T cells
Source: PLoS Pathog. 2020 Aug 6;16(8):e1008752. doi: 10.1371/journal.ppat.1008752 (PMC7433898; doi:10.1371/journal.ppat.1008752)
Supplement: S1 Table — (DOCX) [file ppat.1008752.s005.docx]

**Table S1: Primers used to generate pCG_IRES_BFP expression constructs.**

| **Construct name** | **Primer name** | **Primer sequence** |
| --- | --- | --- |
| PYHIN1 | PYHIN1 XbaI fw | CGTCTAGACCATGGCAAATAACTACAAG |
|  | PYHIN1 C-HA MluI rev | CTACGCGTTAAGCGTAATCTGGAACATCGTATGGGTAGGAACTGCTGGATGG |
| MNDA | MNDA XbaI fw | CGTCTAGACCATGGTGAATGAATACAAG |
|  | MNDA C-HA MluI rev | CTACGCGTTAAGCGTAATCTGGAACATCGTATGGGTAATTAACATTCATTGGTCCTTC |
|  | Remove internal XbaI fw | GCCTGTCTTGACAAACTAATAGAAC |
|  | Remove internal XbaI rev | GTTCTATTAGTTTGTCAAGACAGGC |
| PYHIN1 PYD and linker | PYHIN1 XbaI fw | CGTCTAGACCATGGCAAATAACTACAAG |
|  | PYHIN1 PYD and linker C-HA MluI rev | CTACGCGTTAAGCGTAATCTGGAACATCGTATGGGTACAATGGTTTTAGGCTCTCAG |
| PYHIN1 PYD only | PYHIN1 XbaI fw | CGTCTAGACCATGGCAAATAACTACAAG |
|  | PYHIN1 PYD only C-HA MluI rev | CTACGCGTTAAGCGTAATCTGGAACATCGTATGGGTATTTTAACTTTTCTCTTTTAAGAGTTTC |
| MNDA PYD and linker | MNDA XbaI fw | CGTCTAGACCATGGTGAATGAATACAAG |
|  | MNDA PYD and linker C-HA MluI rev | CTACGCGTTAAGCGTAATCTGGAACATCGTATGGGTACTGGGTTTCCTGATTCG |
| MNDA PYD only | MNDA XbaI fw | CGTCTAGACCATGGTGAATGAATACAAG |
|  | MNDA PYD only C-HA MluI rev | CTACGCGTTAAGCGTAATCTGGAACATCGTATGGGTATTTTGACTTCTCTTTTCGAAG |
| AIM2 PYD and linker | AIM2 XbaI fw | CGTCTAGACCATGGAGAGTAAATACAAGG |
|  | AIM2 PYD and linker C-HA MluI rev | CTACGCGTTAAGCGTAATCTGGAACATCGTATGGGTACTGGGCCACCATCTG |
| AIM2 PYD only | AIM2 XbaI fw | CGTCTAGACCATGGAGAGTAAATACAAGG |
|  | AIM2 PYD only C-HA MluI rev | CTACGCGTTAAGCGTAATCTGGAACATCGTATGGGTATTTCTCCTTCTCCTCCTG |
| IFI16-AIM2 chimeras | IFI16 XbaI fw | CGTCTAGACCATGGGAAAAAAATACAAGAACATTGTTC |
|  | IFI16 PYD+AIM2 linker overhang rev | CGATTTGTATTGCTTATCAACTTTTAACTTTTCTTTTTTAAGAGTTTC |
|  | IFI16 HinA+AIM2 linker overhang fw | GCCTGAACAGAAACAGGCCAAATGTCAGGTAAC |
|  | IFI16 C-HA MluI rev | CTACGCGTTAAGCGTAATCTGGAACATCGTATGGGTAGAAGAAAAAGTCTGGTGAAGTTTCC |
|  | AIM2 linker + IFI16 PYD overhang fw | GAAACTCTTAAAAAAGAAAAGTTAAAAGTTGATAAGCAATACAAATCG |
|  | AIM2 linker + IFI16 HinA overhang rev | GTTACCTGACATTTGGCCTGTTTCTGTTCAGGC |
|  | AIM2 XbaI fw | CGTCTAGACCATGGAGAGTAAATACAAGG |
|  | AIM2 PYD+IFI16 linker overhang rev | GATAGGGCTGGTCCTTTTACTTTCTCCTTCTCCTCCTG |
|  | AIM2 HinC+IFI16 linker overhang fw | GAGAACCCGAAAACAGTGATGGTGGCCCAGC |
|  | AIM2 C-HA MluI rev | CTACGCGTTAAGCGTAATCTGGAACATCGTATGGGTATGTTTTTTTTTTGGCCTTAATAAC |
|  | IFI16 linker+AIM2 PYD overhang fw | CAGGAGGAGAAGGAGAAAGTAAAAGGACCAGCCCTATC |
|  | IFI16 linker+AIM2 HinC overhang rev | GCTGGGCCACCATCACTGTTTTCGGGTTCTC |
| Sp1 C-FLAG | Sp1 HA_to_FLAG fw | GATGATGATAAATGAACGCGTCGGATCCTG |
|  | Sp1 HA_to_FLAG rev | ATCTTTATAATCGAAGCCATTGCCACTGATATTAATG |
| Sp1 ΔID | Sp1 ΔID fw | CCGAGTCAGTCAGGGGGA |
|  | Sp1 ΔID rev | CATGGTCTAGAAGGCCTACG |
| Sp1 ΔTrA | Sp1 ΔTrA fw | TATGTGACCAATGTACCAGTGG |
|  | Sp1 ΔTrA rev | GCCCTGGGAGTTGTTGCT |
| Sp1 ΔTrB | Sp1 ΔTrB fw | AGCAGCAGCAACACCACT |
|  | Sp1 ΔTrB rev | CTGAGTCTGTCCTGAGAGTAC |
| Sp1 ΔCD | Sp1 ΔCD fw | GACAGTGAAGGAAGGGGC |
|  | Sp1 ΔCD rev | GGTCTGCCCCAAGGAAAC |
| Sp1 ΔZnF | Sp1 ΔZnF fw | CAGAATAAGAAGGGAGGCCCAGG |
|  | Sp1 ΔZnF rev | TCCCCCGAGCCCCTTCCT |
| Sp1 ΔDD | Sp1 ΔDD fw | GATTATAAAGATGATGATGATAAATG |
|  | Sp1 ΔDD rev | GTGGGTCTTGATATGTTTTG |
| IFI16 L10A-L11A | L10A-L11A fw | cgaaaAGGATTAGAGGTCATCAATG |
|  | L10A-L11A rev | tcgaaCAATGTTCTTGTATTTTTTTCC |
| IFI16 S27A | S27A fw | cctACTGAGCAACGATTTAAAAC |
|  | S27A rev | ccttAACCATTCTAAAATGATAATCATTG |
