# Supplementary material for: Nuclear PYHIN proteins target the host transcription factor Sp1 thereby restricting HIV-1 in human macrophages and CD4+ T cells
Source: PLoS Pathog. 2020 Aug 6;16(8):e1008752. doi: 10.1371/journal.ppat.1008752 (PMC7433898; doi:10.1371/journal.ppat.1008752)
Supplement: S2 Table — (DOCX) [file ppat.1008752.s006.docx]

**Table S2: Primers and probes used for qRT-PCR****.**

| **Name** | **Sequence** |
| --- | --- |
| qPCR Long LTR fw | GCCTCAATAAAGCTTGCCTTGA |
| qPCR Long LTR rev | GGGCGCCACTGCTAGAGA |
| Long LTR probe | CCAGAGTCACACAACAGACGGGCACA |
| qPCR Nef fw | GGTGGGAGCAGYATCTCGAGA |
| qPCR Nef rev | TGTAAGTCATTGGTCTTAAAGGTACCTGAGG |
| Nef probe | TGCTTCYAGCCAGGCACAAKCAGCATT |
| qPCR PolyA fw | GCCCTCAGATGCTRCATATAA |
| qPCR PolyA rev | TTTTTTTTTTTTTTTTTTTTTTTTTTGAAG |
| PolyA probe | TGCCTGTACTGGGTCTCTCTGGTTAG |
