# Supplementary material for: Nuclear PYHIN proteins target the host transcription factor Sp1 thereby restricting HIV-1 in human macrophages and CD4+ T cells
Source: PLoS Pathog. 2020 Aug 6;16(8):e1008752. doi: 10.1371/journal.ppat.1008752 (PMC7433898; doi:10.1371/journal.ppat.1008752)
Supplement: Attachment — Submitted filename: Bosso et al Rebuttal.pdf [file ppat.1008752.s007.pdf]

Reply to the reviewer`s comments (*in italic letters*)

Reviewer #1: In this study Bosso and colleagues have investigated the antiviral activities of PYHIN proteins with a focus on the less well-characterized proteins MNDA and PYHIN1. The authors provide evidence for a model in which these proteins have broad antiviral activity through interaction with, and perhaps sequestration of, the transcription factor Sp1. The experiments are carefully thought out and performed and the data are in the most part compelling. If the Sp1 interaction business can be sorted out satisfactorily this will be a comprehensive study with good impact.

We thank the reviewer for this positive assessment. As specified below, we solved the background problem in the Sp1/IFI16 Co-IP (revised Figure 9B; previously Figure 7B). Notably, we also obtained additional results in primary HIV-1-infected CD4+ T cells showing that Sp1 and IFI16 are in close proximity in the nucleus (new Figure 4E) and that Sp1 co-immunoprecipitates with endogenous IFI16 (new Figure 4F).

Reviewer #2: The overall quality of this study is poor, relying largely on inhibition of HIV-1 production by overexpressed Pyhin proteins. Several experiments are unconvincing (see below). The study is also not particularly novel, as the same group recently published (in a significantly stronger paper) that one of this group of pyhin proteins inhibits HIV-1, via the same mechanism (Sp1 sequestration) as reported here.

We respect this reviewer`s opinion, but like to point out that we also performed comprehensive expression and KD analyses in primary human macrophages. The results on PYHIN1 and MNDA as well as many mechanistic data are novel. Perhaps most importantly, we included new data demonstrating for the first time that IFI16 restricts transmitted-founder HIV-1 strains in primary CD4+ T cells and supporting that IFI16 and Sp1 interact in the nucleus of primary viral target cells (new Figures 4, S3).

Reviewer #3: The authors reported in a recent paper that IFI16, an interferon-inducible member of the family of pyrin and HIN domain containing (PYHIN) proteins, "restricts HIV-1 independently of immune sensing by binding and inhibiting the host transcription factor SP1". They also reported that SP1 binding and anti-HIV-1 activity depend on the IFI16 pyrin domain (PYD) and its nuclear localization, but not on the DNA-binding HIN domain.

In the present study, the authors extend these findings by showing that the PYDs of other human PYHIN proteins also bind SP1 and inhibit HIV-1, as long as they are localized to the nucleus. Additionally, native human PYHIN proteins MDNA and PYHIN1 but not AIM2 are shown to be nuclear and to inhibit HIV transcription when overexpressed in 293T cells. Furthermore, knockdown experiments indicate that PYHIN1 and MNDA restrict HIV-1 in primary human macrophages. Altogether, the evidence presented suggests that nuclear PYDs, and in some cases the native proteins, inhibit HIV and some other viruses, as well as retrotransposons, by reducing the availability of the SP1 transcription factor. While this is an interesting model, most of the data on HIV were obtained with a rather artificial experiment setup, and the manuscript would benefit from additional evidence establishing relevance of the observations in more physiologically relevant HIV target cells.

We thank this reviewer for the constructive comments. The HEK293T cell-based assays are highly useful for mechanistic studies. However, we agree that key findings should be confirmed in relevant HIV target cells and had therefore performed experiments in primary macrophages (Figure 3). Meanwhile, we established two different methods to reduce IFI16 expression in primary CD4+ T cells. Our new data demonstrate that depletion of IFI16 strongly increases infectious yield of HIV-1 strains that are sensitive to IFI16 in transient expression assays in primary CD4+ T cells (new Figures 4 and S3). In addition, we show that IFI16 is in close proximity to Sp1 in the nucleus of CD4+ T lymphocytes (new Figure 4E) and that Sp1 co-immunoprecipitates with endogenous IFI16 (new Figure 4F). Thus, we confirmed key findings of our study in the major target cells of HIV-1.

Part II – Major Issues: Key Experiments Required for Acceptance

Reviewer #1: 1. A key experiment presented in Fig7 is the interaction between IFI16 and Sp1 full length and deletions by co-immunoprecipitation. Unfortunately the Sp1 protein and most of its fragments clearly bind the beads in the absence of HA-Tagged IFI16. This is problematic because the pattern of binding with and without the immunoprecipitated IFI16 is almost identical. Note that the deltaZNF Sp1 fragment doesn't bind the beads and doesn't co-precipitate with IFI16. The only specific interaction appears to be the deltaCD Sp1 fragment, which is only present in the co-precipitate, and not in the absence of tagged IFI16 but the authors don't specifically discuss this. However, given this band is the weakest co-precipitated protein, this may simply be below the detection limit in the absence of IFI16. Basically, this experiment is not compelling enough to hang the whole story on including the title of the paper.

It is true that the precipitated bands are denser but the BFP bands are slightly denser on the right. Unfortunately, the specificity is pattern pretty much identical with and without the HA-tagged IFI16.

I'm not sure how to resolve this. The authors might take this out and revise their conclusions. They might sort the conditions out so the co-immunoprecipitation is specific. Unfortunately, if the protein you're working on binds the beads, then one can't really use this technique and conditions to measure protein-protein interactions.

The reviewer raised an important point. To address it, we optimized the conditions for the co-IP experiments by including a pre-clearing step to eliminate the unspecific background. Our new results confirm that IFI16 and Sp1 interact and that deletion of the Zinc finger motif disrupts this interaction (revised Fig 9B, previously 7B). To further address this, we performed proximity ligation and Co-IP assays in primary CD4+ T cells and the results from both experiments support an interaction between Sp1 and IFI16 in the nucleus (new Figs 4E, 4F).

2. bottom page 10. Do I understand correctly that the authors think that AIM2 can activate ASC specks in the nucleus when fused with the IFI16 NLS. I may have misunderstood this but they say that the AIM2-IFI16 chimera goes into the nucleus and still activates ASC speck formation. I would interpret this as AIM2 activating ASC specks in the cytoplasm as usual due to remaining cytoplasmic AIM2-IFI16 chimera or newly synthesized AIM2-IFI16 before it goes into the nucleus. The discussion on line 317 seems to imply that in this case the ASC specks might be in the nucleus, which I doubt. If they think the specks might be in the nucleus in this case, they could easily test that by IF, which they should do if that's what they think. Please clarify the interpretation of this chimera result. No more experiments are necessarily required for this point.

We agree that it is well possible (and perhaps even likely) that AIM2 with the NLS nucleated inflammasomes while it was transiently localized in the cytosol (e.g. shortly after translation). The main point is that IFI16 with its NLS still does not nucleate specks, meaning that the presence of the NLS cannot explain why IFI16 is not able to nucleate inflammasomes. We rephrased the text for clarity (lines 252-263) and show primary IF data for the ASC specks (new Figure 7B).

Reviewer #2: 1. If the pyhin proteins inhibit transcription by sequestering SP1, he authors need to provide some explaination for the fact that the CMV promoter, which is activated by Sp1, appears resistant to the pyhin proteins (Figure 1D).

This seems a bit outside of the scope of the present study. Nonetheless, we performed titration analyses demonstrating that alterations in the levels of NF-kB, but not Sp1 affect the activity of the CMV promoter, while both affect the HIV-1 promoter (new Figure S1). The HCMV major immediate-early promoter (*MIEP) is known to be activated by a wide variety of host transcription factors and we did not find a study*

demonstrating dependency on Sp1. Our results clearly demonstrate that variations in Sp1 levels have has no significant effect on its activity in the present experimental set-up.

2. The RNAi knockdown experiment shown in Figure 3/Figure S1 is not compelling. The knockdowns are extremely variable, often not specific for the targeted protein and some knockdowns appear to affect cell viability (total cell protein). Some knockdowns even give the appearance of upregulating other Pyhin proteins, in an inconsistent manner. Overall this is a very unconvincing dataset. Since this experiment is the only one in the paper that does not involve overexpressed Pyhin proteins, this reviewer is very doubtful that pyhin proteins restrict HIV-1 infection when present at endogenous levels.

It is correct that the results varied to some extent. However, this is not unusual when working with primary cells from different donors. To ensure that our results are valid, we examined macrophages from a total of nine different donors. On average, knockdown of the nuclear Pyhin proteins by 50% to 80% increased infectious virus yield after 6 days ~20-fold, while no significant increase was observed after treatment with control siRNA or knockdown of AIM2. Differences were significant and the results conclusive. Importantly, we now demonstrate that KD of IFI16 in primary CD4+ T cells also strongly increases infectious HIV-1 production (new Figures 4; S3).

3. The magnitude of the antiviral effect displayed by these proteins is modest (3 to 5-fold inhibition in most experiments), even when grossly overexpressed by transient transfection in 293T cells. Stable expression of these proteins in a T-cell line should be able to prevent HIV-1 replication in a viral growth assay, if the authors model is correct.

In our opinion a 3- to 5-fold effect is not modest. More importantly, KD of endogenous IFI16, PYHIN1 and MNDA in macrophages frequently increased HIV-1 production by more than one order of magnitude. We now show that partial KO or KD of IFI16 in primary CD4+ T cells also increases infectious virus yield up to 20-fold. Thus, the inhibitory effect on primary HIV-1 strains in primary human cells is very strong.

4. Figure 6 shows great variation in the sensitivity to pyhin proteins among HIV-1 strains which is unexplained. This result suggests that is it trivially easy for HIV-1 to escape the antiviral effects of pyhin proteins, which isn't the expected property of a genuine restriction mechanism.

We have previously shown that the susceptibility to IFI16 maps to the LTR and correlates with virus dependency on Sp1 for transcription (Hotter et al., 2019). We also found that highly prevalent subtype C viruses are less dependent on Sp1 and largely resistant to IFI16 and now confirm this in CD4+ T cells. Having observed a commonly shared mechanism, it is not surprising that subtype C viruses are less inhibited by IFIX and MNDA as well. It is well established that HIV can escape many major restriction mechanisms and the changes conferring resistance against nuclear Pyhin proteins may come at a cost. Our results obtained using TF viruses from different clades demonstrate that thus far only subtype C HIV-1 strains are capable of evading this restriction mechanisms; thus, as now described in the revised discussion section (lines 392-412) evasion is certainly not "trivially easy" and/or most certainly comes at a cost.

5. The coprecipitation assays in Figure 6D and 7B showing that pyhin proteins bind to Sp1 are unconvincing – Controls are inadequate, there is a significant signal in the control lanes, and both active and inactive pyhin proteins apparently coprecipitate Sp1.

As specified in the response to point 1 of reviewer 1, we optimized the Co-IP assay (revised Figure 9B) and confirmed key findings in primary CD4+ T cells (Figures 4, S3). It is not unexpected that AIM2 interacts with Sp1 in cellular extracts since enforced re-localization of AIM2 to the nucleus is sufficient to render it antivirally active (Figure 6C). In the Co-IP protocol, cells are homogenized and thus cytoplasmic AIM2 comes into contact with nuclear Sp1.

6. The experiments in Figure 6 are extremely superficial. Based on their varying susceptibility to inhibition by overexpressed Pyhin proteins it is claimed that the other viruses used in these experiments may be inhibited by different mechanisms. If so, then these experiments are irrelevant to the current study.

We agree that the results only provide first insights and hope that they will simulate further studies. Nonetheless, the different activities of the various mutants clearly indicate different underlying mechanisms. Thus, we decided to keep these data but clearly mention their preliminary nature.

Reviewer #3: 1. The conclusions regarding the mechanism of HIV inhibition (interference with Sp1-dependent gene transcription; competition of PYDs with DNA for SP1 binding) are entirely based on overexpression in 293T cells. The manuscript could be strengthened by evidence that this also occurs in a cellular context that is more relevant for HIV-1. 293T cells are not physiologically relevant target cells, and typically express unusually high protein levels upon transfection.

To address this valid point, we performed proximity ligation assays (PLA) showing that endogenous Sp1 and IFI16 are in close proximity in the nucleus of primary CD4+ T cells (new Figure 4E). In addition, we found that Sp1 and IFI16 can be co-precipitated in primary cells (new Figure 4F).

2. The biological relevance of the study hinges on the primary macrophage experiments (Fig. 3), for which only a single control siRNA was used. In the experience of this reviewer, certain "control siRNAs" can have significant effects on infectious HIV-1 yields, and the authors should exclude this possibility.

We have previously verified that this control siRNA did not affect infectious HIV-1 yield. Notably, the AIM2 siRNA also did not enhance infectious virus yield. Perhaps most importantly, we now confirmed that IFI16 restricts HIV in primary CD4+ T cells using two different approaches to attenuate IFI16 expression (new Figs 4, S3).

3. Fig. 5C: Why did the "IFI16+AIM2 linker" construct increase HIV-1 expression in a dose-dependent manner? This enhancement was almost as pronounced as the inhibition by native IFI16. Clearly, the AIM2 linker did not just disrupt the function of IFI16, as the authors state (line 192).

We agree that it might affect other functions. The enhancing effect was only observed on GFP expression but not in the infectious HIV-1 yield assay (now Fig 6C). We do not have experimental data that explain the modest enhancing effect on GFP expression and mention this in the revised version (line 229-231).

Part III – Minor Issues: Editorial and Data Presentation Modifications

Reviewer #1: 3. line 89, add "when over-expressed" in 293T cells, for clarity. *Done.*

4. Figures 1D, 5C, use symbols that are more different for clarity. *Changed as suggested.*

5. Figure 5F, can we see some IF of the specks? Do they look like we expect?

We now show IF images of the specks (Figure 7B). They look as expected.

6. In Figure 2 the authors say they made a standard curve with an IFN titration. Can we see the data plotted as IFN units which would be more meaningful. If this isn't possible, that's fine, but if they've gone to the trouble to drive the IFN values, I'd show them.

We now show the curve for comparison in the revised Fig 2. It validated the assay but there are not enough data points for calculation of IFN units. Thus, we prefer to show the primary luciferase reporter values.

7. Do the authors think that AIM2 antiviral activity is through binding and titrating Sp1? Is this expected for a protein that doesn't normally go into the nucleus? Can they clarify this point?

Yes, this is what we think and it came as surprise. However, it is known that the pyrin domain of all PYHIN proteins are involved in interactions with various target proteins and it has been reported that some amino acid residues involved in these interactions are generally conserved (Morrone et al., 2014). Following up on this, we now show that residues L10/L11 in IFI16 that are conserved in AIM2 (Fig S4C) are critical for antiviral activity (Fig 9D). Thus, the interaction of AIM2 with Sp1 might reflect a broader generally conserved ability for protein interactions but this needs further study.

8 line 266 the authors are not investigating the effect of HBV causing 250 million infections, reword for clarity. *Done*

Reviewer #2: (No Response)

Reviewer #3:

1. Page 8, line 157; Fig. S1B: The statement that "the levels of MNDA expression were significantly reduced in virally infected cultures" is difficult to reconcile with the data shown in Fig. S1B.

On average, the reduction was ~40%. As specified in the methods section, the bands were quantified and normalized to GAPDH. The reduction may not be well visible but was clearly identified by quantitative analyses.

2. Fig. 3C: The Y axis seems to be mislabeled (% of ctrl siRNA). According to the text, the figure shows the effects of HIV-1 infection on protein expression levels.

The numbers provide the percentage of protein levels compared to those detected after treatment with the control siRNA (100%). We agree that this might be misleading, Thus, we changed the label and modified the figure legend.

3. Page 10, top: a subheading should be used for the section on inflammasome assembly. *Added*

4. Fig. 6F: The IFI16-AIM2 linker construct should also be included here. *Done (new Fig 8G)*

5. Page 12 and 13: Fig. 7A-G are all mislabeled (should be Fig. 8A-G).

We apologize for this mistake and have corrected it.
